# Latent Membrane Protein 1 Is a Novel Determinant of Epstein-Barr Virus Genome Persistence and Reactivation

**DOI:** 10.1128/mSphereDirect.00453-17

**Published:** 2017-11-08

**Authors:** Elizabeth A. Caves, Rachel M. Butch, Sarah A. Cook, Laura R. Wasil, Chen Chen, Yuanpu Peter Di, Nara Lee, Kathy H. Y. Shair

**Affiliations:** a Cancer Virology Program, University of Pittsburgh Medical Center, Pittsburgh, Pennsylvania, USA; b Department of Microbiology and Molecular Genetics, University of Pittsburgh, Pittsburgh, Pennsylvania, USA; c Department of Environmental and Occupational Health, University of Pittsburgh, Pittsburgh, Pennsylvania, USA; University of Michigan—Ann Arbor; Penn State University College of Medicine; University of Wisconsin–Madison

**Keywords:** Epstein-Barr virus, gammaherpesvirus, pathogenesis

## Abstract

Epstein-Barr virus (EBV) is a ubiquitous gammaherpesvirus that persistently infects humans, with nearly 95% seropositivity in adults. Infection in differentiating epithelia is permissive, but EBV-associated nasopharyngeal carcinoma (NPC) tumors harbor a clonal and nonproductive latent infection. However, in explanted NPC cultures and epithelial cell lines, episomal EBV genomes are frequently lost. The resulting unstable infection has hampered efforts to study the determinants of EBV persistence and latency in epithelial oncogenesis. The EBV nuclear antigen 1 (EBNA1) protein is required for tethering EBV episomes to cellular DNA and for mitotic segregation to daughter cells. Expression of EBNA1 does not ensure faithful partitioning of EBV episomes or replicons, suggesting that additional regulatory mechanisms have yet to be elucidated. The EBV latent membrane protein 1 (LMP1) is an oncogenic signaling protein expressed in latent and lytic cycles. This study identified that LMP1 contributes to the loss of EBV genomes in latently infected cells and promotes differentiation-induced lytic replication in a polarized air-liquid interface (ALI) culture model. Deletion of LMP1 in recombinantly infected 293 cells promoted the retention of EBV genomes in passaged cells, which was in part localized to a conserved PXQXT motif in the C-terminal signaling domain (CTAR1). Additionally, knockdown of LMP1 in the recombinantly infected NPC cell line HK1 resulted in decreased induction of lytic proteins and infectious EBV titers. These findings are consistent with the hypothesis that in epithelial infections, regulation of LMP1 mechanisms may be a determinant of infection outcome and a potential risk factor for EBV persistence in preneoplastic cells.

**IMPORTANCE** Latent membrane protein 1 (LMP1) is a constitutively active oncogenic signaling protein encoded by Epstein-Barr virus (EBV). Despite monoclonal infection in cases of nasopharyngeal carcinoma (NPC), it has been difficult to reconcile the heterogeneous LMP1 protein levels detected in tumor cells. The LMP1 protein is a pleiotropic signaling protein with oncogenic potential. Findings from this study are consistent with the hypothesis that LMP1 has a role distinct from that of oncogenesis that facilitates the viral life cycle by promoting an unstable but productive infection in differentiating epithelia.

## Abstract (precis)

Latent membrane protein 1 (LMP1) is a constitutively active oncogenic signaling protein encoded by Epstein-Barr virus (EBV). Despite monoclonal infection in cases of nasopharyngeal carcinoma (NPC), it has been difficult to reconcile the heterogeneous LMP1 protein levels detected in tumor cells. The LMP1 protein is a pleiotropic signaling protein with oncogenic potential. Findings from this study are consistent with the hypothesis that LMP1 has a role distinct from that of oncogenesis that facilitates the viral life cycle by promoting an unstable but productive infection in differentiating epithelia.

## INTRODUCTION

Two factors likely impact the outcome of Epstein-Barr virus (EBV) infection in differentiating epithelia to favor the association of latent EBV infection in epithelial cancers: (i) the ability to retain EBV episomes in replicating cells of the basal epithelium and (ii) the suppression of differentiation-induced lytic reactivation to promote a nonproductive latent infection (1–5). Although the EBV nuclear antigen 1 (EBNA1) protein is required for tethering EBV episomes to cellular genomic DNA and for mitotic segregation to daughter cells, expression of EBNA1 does not ensure the faithful partitioning of EBV episomes or reporter replicons, suggesting that additional factors regulate the retention of EBV genomes in replicating epithelial cells (6–9). The inefficient retention of EBV genomes in replicating cells has added to the challenge of establishing persistently infected EBV cell lines either by *de novo* infection or by cultivation of explanted nasopharyngeal carcinoma (NPC) cells, both of which eventually lead to the loss of infection in the absence of recombinant selection. LMP1 is one of four (EBNA1, LMP1, LMP2A, LMP2B) latent, protein-coding transcripts expressed in NPC tumors (10). Although LMP1 is a latent transcript expressed in type II and III latencies, it is also expressed in the lytic cycle (11). LMP1 protein is a ligand-independent, constitutively active, oncogenic signaling molecular, but its role in viral pathogenesis and its potential effects on the viral life cycle have not been fully elucidated (12, 13).

As a double-stranded DNA virus, EBV genome sequences are well conserved and phylogenetically segregate into distinct types, but the latent genes are more likely to contain sequence variation (14). There are seven recognized LMP1 sequence variants defined as LMP1 strains, but the major signaling domains termed C-terminus-activating regions 1 and 2 (CTAR1 and -2) are conserved in all LMP1 strains (10, 13, 15). All LMP1 strains circulate in the peripheral blood and saliva of asymptomatic carriers, but China1 is the predominant LMP1 strain detected in NPC tumors (16, 17). Although the detection of a prevalent LMP1 China1 strain in NPC might be due to enhanced immune evasion properties, it is also possible that LMP1 strains may be distinguished by their role in EBV pathogenesis (18).

NPC tumors are defined by a monoclonal and latent EBV infection (2, 19). The prevailing dogma is that the establishment of a latent reservoir and continual propagation of cells that efficiently retain segregated episomal EBV genomes are likely preneoplastic conditional events preceding the establishment of EBV-associated tumors. The limited availability of clinical biopsy specimens that capture preinvasive lesions and the experimental obstacles in establishing *de novo*-infected cell lines has led to many challenges in elucidating the early determinants of oncogenesis. Using recombinant genetics, this study examines EBV-bacterial artificial chromosome (BAC) viruses and mutants in recombinantly infected 293 cell lines to test the potential role of LMP1 in EBV genome retention in serially passaged replicating cells. Contrary to the prediction that LMP1 promotes a persistent infection, deletion of LMP1 in an EBV-BAC resulted in more-stable retention of latent EBV genomes in passaged 293 cells. Additionally, an NPC cell line (HK1) recombinantly infected with an EBV Akata strain was used to test the efficiency of lytic reactivation in polarized air-liquid interface (ALI) cultures. Differentiation by lifting epithelial monolayers to an air-liquid interface is a physiological stimulus for inducing lytic reactivation (11, 20, 21). Knockdown of LMP1 in EBV Akata-infected HK1 cells cultured in the air-liquid interface suppressed lytic reactivation, as demonstrated by decreased induction of lytic proteins and infectious virus production. These findings establish a new paradigm for LMP1 in permissive epithelial cell infection that may promote lytic viral spread but is incompatible with the persistent latent infection observed in latent NPC tumors.

## RESULTS

### LMP1 interferes with EBV genome persistence.

**FIG 1:**
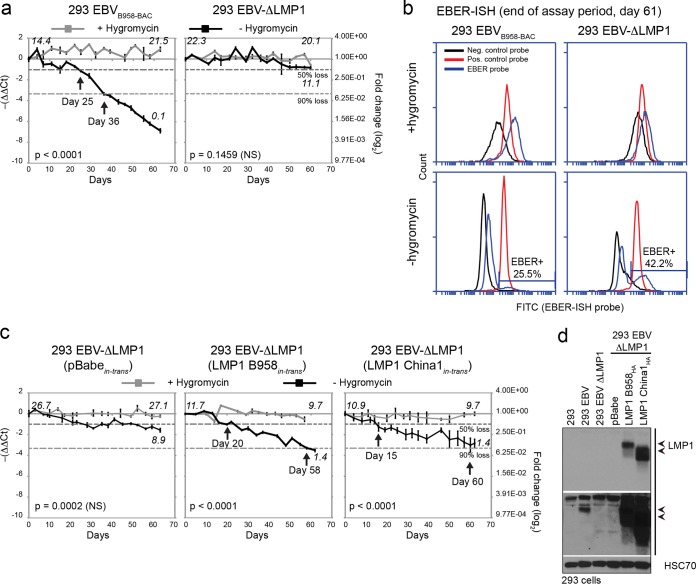
Deletion of LMP1 promotes the retention of EBV genomes in 293 cells. (a) Quantitative PCR measuring the loss of EBV genomes in serially passaged 293 cells infected with a B958 strain of wild-type EBV-BAC or an LMP1 deletion mutant. (b) Flow cytometry analysis of EBER-ISH measuring the loss of EBV-infected cells was carried out in a repeat experiment. (c) Quantitative PCR measuring the loss of EBV genomes in 293 cells infected with an LMP1 deletion mutant complemented (in *trans*) with an expression vector (pBabe) for LMP1 B958 or China1 strain cDNA. (d) Immunoblot analysis for LMP1 expression in EBV-infected and *trans*-complemented 293 cell lines. EBV genome copy numbers per cell at the beginning and end of each assay as defined by comparison to Namalwa cells are denoted in italics. NS, not significant between cells with and without hygromycin selection (*P* = 0.0001).

In order to examine the effect of LMP1 on the retention of EBV genomes in replicating cells, an EBV-BAC recombinant (B958 strain) and an LMP1 deletion mutant (ΔLMP1 strain) were analyzed in infected 293 cells that are stably maintained by the BAC-introduced hygromycin resistance gene (22, 23). Under conditions of induced reactivation, these 293 cells can produce infectious virus by transfection of the EBV immediate early and late genes (Z/gB). However, under uninduced conditions, Southern blot analysis did not detect linear replicating genomes, and the absence of titratable infectious virus supports the finding that infection in 293 cells is primarily latent (see Fig. S1 in the supplemental material) (23). In the absence of evidence to indicate lytic reactivation, the retention of episomal EBV genomes was therefore determined in uninduced 293 cell lines. The BAC recombinant EBV genomes encode green fluorescence protein (GFP), but expression from the ectopic promoter can be stochastically silenced and is not a good surrogate for determining infection loss. Additionally, measuring the loss of infection depends on numbers of EBV copies per cell, which are highly varied between cell lines and may take a long period of time (beyond several months) to determine (Fig. S2). Therefore, quantitative PCR (qPCR) for the EBV BALF5 gene, normalized to cellular GAPDH (glyceraldehyde-3-phosphate dehydrogenase), was used to determine the loss of EBV genomes in serially passaged cells grown in the absence or presence of recombinant selection. Removal of hygromycin selection resulted in a 50% decrease in EBV genomes by 25 days and a 90% loss in the 36-day serial culture (Fig. 1a). In contrast, cells grown in the presence of selection maintained a fluctuating but consistent number of EBV genomes over time, which may reflect the copy number variation inherent in a cell population (14 to 21 averaged EBV genome copies/cell). Despite the variability, all cells grown in the presence of selection retained EBV infection as determined by Epstein-Barr encoding region *in situ* hybridization (EBER-ISH), which detects the abundant noncoding EBER transcripts expressed in all EBV-infected cells (Fig. 1b) (6, 24). However, the decrease to 90% in the absence of selection corresponded with a decrease in EBER-positive cells to 25.5% in a repeat experiment, further supporting infection loss (Fig. 1b) (4, 6). Unlike in the wild-type EBV-BAC infection, cells infected with EBV-ΔLMP1 more readily retained EBV genomes, corresponding with 42.2% EBER positivity at the end of the assay period (Fig. 1a and b). The retention of EBV genomes in EBV-ΔLMP1-infected cells could be reversed by the stable expression of LMP1 in *trans*, reaching 50% EBV genome loss by 20 days and 90% loss by 58 days, while cells expressing the retroviral vector control (pBabe) remained relatively stable at no more than a 50% loss, similar to what occurred with the LMP1 deletion mutant (Fig. 1c).

10.1128/mSphereDirect.00453-17.1 FIG S1 Southern blot analysis of episomal and reactivated linear EBV genomes. (a) Schematic of BamHI-digested episomal and linear EBV genomes as detected by an LMP1 (265-bp) or Xho1a (1.9-kb) probe leftward of the terminal repeats (TR). Episomal EBV genomes have fused termini and are detected as a singular hybridized BamHI-digested band. Reactivated linear genomes are not fused and are detected as multiple hybridized BamHI-digested bands separated by 500-bp increments corresponding to the number of terminal repeats. (b and c) Southern blot for BamHI-digested DNA hybridized to a biotinylated LMP1 DNA probe (b) or a more sensitive random-primed radiolabeled Xho1a (1.9-kb) DNA probe (c). Samples with weak detection (boldface) were repeated with double the amount of BamHI-digested DNA (8 μg) and hybridized to the Xho1a probe. Z/gB, transfected with BZLF1 (Z) and BALF4 (gB) to induce lytic reactivation. A 1:4 dilution of BamHI-digested DNA was loaded to reduce signal intensity. Download FIG S1, EPS file, 6 MB. Copyright © 2017 Caves et al. 2017 Caves et al. This content is distributed under the terms of the Creative Commons Attribution 4.0 International license.

10.1128/mSphereDirect.00453-17.2 FIG S2 Quantitation of EBV genomes by averaged copy numbers per cell. Quantitative PCR of EBV genomes relative to copy numbers defined in Namalwa and Raji cells at day 0 (D0) and at the end of each assay in the presence or absence of selection (hygromycin, +H and −H; neomycin, +N and −N). Download FIG S2, EPS file, 1.9 MB. Copyright © 2017 Caves et al. 2017 Caves et al. This content is distributed under the terms of the Creative Commons Attribution 4.0 International license.

Stable transduction of the NPC patient-prevalent China1 strain, in addition to the LMP1 B958 prototypic strain, was examined for the disruption of EBV genome retention in EBV-ΔLMP1-infected cells. In the absence of hygromycin selection, EBV genome loss was restored by transcomplemented expression of LMP1 China1, reaching 50% EBV genome loss by 15 days and 90% loss by 60 days (Fig. 1c). These data indicate that EBV genome loss is a common property conferred by both the B958 and China1 strains and suggest that it may localize to a conserved LMP1 signaling domain.

**FIG 2:**
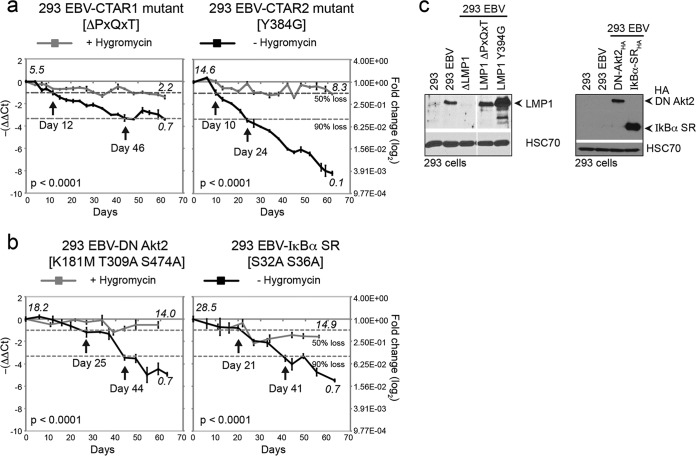
Mapping of LMP1 signaling motifs and cellular pathways. Quantitative PCR to measure the loss of EBV genomes in 293 cells infected with LMP1 signaling mutants (ΔPXQXT and Y384G) (a) or a wild-type EBV-BAC expressing an Akt isoform 2 dominant negative (DN) (K181M T309A S272A) or an IκBα (S32A S36A) superrepressor (b). (c) Immunoblot analysis for the expression of LMP1 and HA-tagged proteins in EBV-infected and stably transduced 293 cell lines. EBV genome copy numbers per cell at the beginning and end of each assay as defined by comparison to Namalwa cells are denoted in italics. NS, not significant between cells with and without hygromycin selection (*P* = 0.0001).

**FIG 3:**
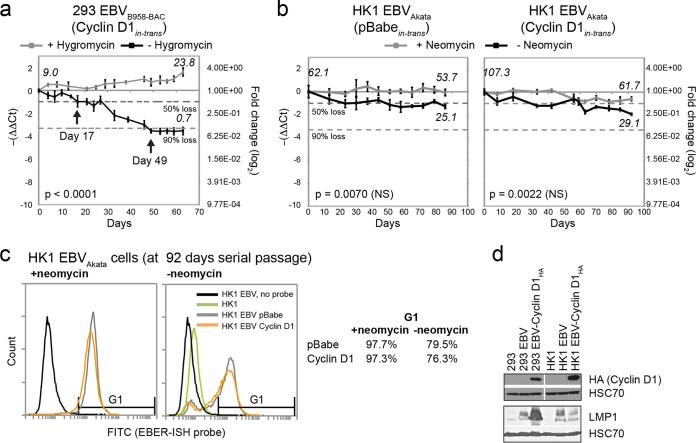
Overexpression of cyclin D1 does not stabilize EBV genomes. Quantitative PCR of EBV genomes in 293 cells infected with the B958-BAC strain of EBV (a) and HK1 cells infected with the Akata strain of EBV (b) stably expressing cyclin D1. (c) Flow cytometry analysis of EBER-ISH-probed HK1 cells. G1 represents the EBER-ISH-positive gate set by the HK1 EBV (no probe) and HK1 uninfected-cell negative controls. (d) Immunoblot analysis for the expression of LMP1 and HA-tagged cyclin D1 in stably transduced 293 and HK1 cell lines. EBV genome copy numbers per cell at the beginning and end of each assay as defined by comparison to Namalwa cells are denoted in italics. NS, not significant between cells with and without selection (*P* = 0.0001).

Expression of LMP1 in EBV-BAC-infected and transcomplemented cell lines was determined by immunoblotting (Fig. 1d). It is possible that the higher LMP1 expression levels detected in *trans-*complemented cells may affect the variable EBV copy numbers compared to those of cells expressing wild-type LMP1 levels (Fig. 1d). EBV genomes were also quantified at the beginning and end of each assay by comparison to two standardized EBV-infected Burkitt lymphoma cell lines with known copy numbers: 45 to 50 averaged episomal copies in Raji cells and 2 integrated copies in Namalwa cells. These values are summarized in Fig. S2 in the supplemental material, and the values from Namalwa comparisons are also displayed in the corresponding qPCR plots (Fig. 1 to 3). Notably, cells not expressing LMP1 (ΔLMP1 and ΔLMP1-pBabe cells) displayed the highest numbers of copies per cell. In comparison, cells stably overexpressing LMP1 (ΔLMP1-LMP1 B958 and ΔLMP1-LMP1 China1 cells) had the lowest numbers of EBV copies per cell (Fig. 1a and c). This is consistent with the notion that LMP1 interferes with the retention of EBV genomes.

### **The** PXQXT** motif in CTAR1 may contribute to the loss of EBV genomes.**

The conserved signaling domains of LMP1 recruits tumor necrosis factor receptor (TNFR)-associated factor (TRAF) and TNFR-associated death domain (TRADD) adapter proteins that activate cellular signaling pathways, including Akt and NF-κB (13). The CTAR1 domain activates Akt and canonical and noncanonical NF-κB pathways, but CTAR2 is the major activator of canonical NF-κB. To determine if the CTAR1 and CTAR2 domains contribute to EBV genome loss, 293 cells infected with CTAR1 or CTAR2 motif mutants were analyzed. Deletion of the PXQXT TRAF-binding motif in CTAR1 (ΔPXQXT), but not mutation of the CTAR2 residue (Y384G) critical for NF-κB activation, resulted in a more progressive loss of EBV genomes, stabilizing at a 90% loss by 46 days (Fig. 2a). However, the effect of EBV genome retention was not as enhanced as in the LMP1 deletion mutant, suggesting that additional LMP1 residues are likely involved. The Akt and NF-κB pathways are activated by the PXQXT motif, and the suppression of these pathways by stable expression of a dominant negative Akt or IκBα superrepressor also resulted in a more progressive loss of EBV genomes, reaching 90% loss by 44 and 41 days, respectively (Fig. 2b). However, the continued loss of EBV genomes beyond 90% indicates that the Akt and canonical NF-κB signaling pathways only partially contribute to the phenotype observed by the loss of the PXQXT signaling motif (Fig. 2b). Immunoblotting detected the expression of LMP1 signaling mutants and hemagglutinin (HA)-tagged Akt and IκBα superrepressor constructs (Fig. 2c). These results support the finding that the interference of EBV genome persistence localizes in part to the CTAR1 PXQXT signaling motif but that mechanisms additional to the activation of Akt and canonical NF-κB pathways may be involved.

### Cyclin D1 does not rescue the loss of EBV genomes.

*De novo* EBV infection in epithelial cells triggers cell cycle arrest, but overexpression of cyclin D1, as occurs in NPC tumors, can overcome EBV-induced arrest and promote the outgrowth of recombinantly infected nasopharyngeal cell lines (25). It is plausible that cyclin D1 may facilitate the outgrowth of EBV-infected cells by promoting the retention of EBV genomes. To investigate further, 293 cells infected with the EBV-BAC recombinant were transduced to stably express HA-tagged cyclin D1 and analyzed for the loss of EBV genomes in the absence of selection. Despite the expression of cyclin D1, EBV genomes decreased to 50% by 17 days and to 90% by 49 days (Fig. 3a). Unlike with 293 cells, *de novo* infection of nasopharyngeal cell lines is inefficient and requires the expression of cyclin D1 to encourage the outgrowth of EBV-infected clones (5, 25). To account for potential differences in cell lines and EBV strains, the effect of cyclin D1 was also analyzed in the NPC cell line HK1, infected with a recombinant EBV-Akata strain encoding neomycin resistance (26). After 70 days of serial passage, comparison of EBV genomes by qPCR did not result in a notable difference in cells grown in the presence or absence of neomycin selection (Fig. 3b). This may be due to the high EBV copy numbers (~50 to 100 copies/cell) in the HK1 EBV-Akata cell line or to a more gradual loss of EBV-infected cells, which can take 6 months (27). As an alternative, cells were stained by EBER-ISH to compare the numbers of infected cells lost. At 3 months (92 days) of serial passage, HK1 EBV-Akata cells stably expressing cyclin D1 resulted in a 21% reduction in EBER positivity (76.3% EBER-ISH^+^) compared to that of cells grown in the presence of neomycin (97.3% EBER-ISH^+^) (Fig. 3c). This reduction was comparable to the 18.2% loss (97.7% versus 79.5% EBER-ISH positivity) in pBabe-transduced vector control cells (Fig. 3c). Stable expression of cyclin D1 was detected by immunoblotting for the HA tag in transduced 293 and HK1 EBV-infected cell lines, and although the level of LMP1 was increased by cyclin D1 expression in 293 cells, the same did not occur in HK1 EBV-infected cells (Fig. 3d). These data support the conclusion that overexpression of cyclin D1 is not sufficient to promote the stable retention of EBV genomes or infection in established recombinant cell lines.

### LMP1 contributes to permissive infection in differentiating epithelia.

**FIG 4:**
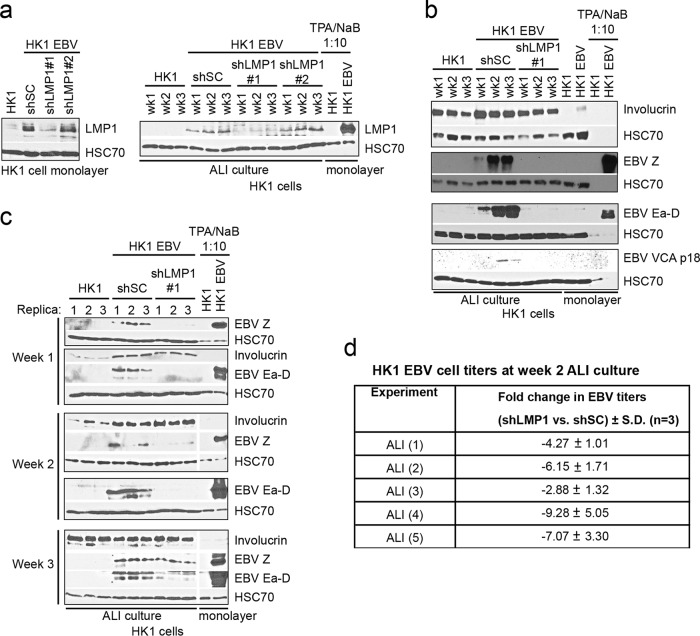
LMP1 contributes to permissive infection in differentiating epithelia. Immunoblot analysis of HK1 cells stably expressing LMP1 shRNAs (shLMP1#1 and shLMP1#2) or a nontargeting sequence (shSC) for knockdown of LMP1 (a), the induction of differentiation and EBV reactivation markers in week 1 to 3 ALI culture (b), and replica experiments (c). (d) Comparison of EBV titers harvested from HK1 cell lines stably transduced with LMP1 shRNA or nontargeting shRNA at week 2 of ALI culture, with titers determined by the green Raji unit assay. Fold changes were calculated relative to HK1 EBV (shSC) cells, averaged from three replica titrations, and compared in five independent experiments. TPA/NaB, tetra-decanoyl phorbol-13-acetate–sodium butyrate reactivation control lysate loaded at a 1:10 dilution.

After a transient period of reactivation, epithelial cells infected with EBV are latent and do not spontaneously reactivate (5, 28). Therefore, the loss of EBV genomes in monolayer culture occurs in the absence of productive replication. However, *in vivo*, EBV-infected polarized epithelial cells are susceptible to differentiation-induced lytic reactivation (20, 29). In comparison to lymphocytes, in which EBV is commonly reactivated with chemical agents such as histone deacetylase (HDAC) inhibitors, airway epithelial cells differentiate when cultured at the air-liquid interface (ALI) on transwell membranes, and this method recapitulates EBV reactivation in polarized epithelia (21, 30). The air-liquid interface culture method is similar to the organotypic raft culture method previously used to induce productive EBV replication in primary gingival and tonsillar epithelial cells and keratinocyte cell lines (20, 29). Conditions favoring latency, in addition to retaining EBV genomes, likely support persistent infection in NPC tumors. While the EBV immediate early switch proteins are necessary for lytic reactivation, it is not clear which EBV proteins may be regulated to promote latency (29). LMP1 is expressed in latency but is also detected as a lytic transcript (Fig. 4a) (11). To determine if LMP1 contributes to differentiation-induced EBV reactivation and infectious-virus production, EBV-infected HK1 cells were analyzed in ALI cultures and compared to LMP1 short hairpin RNA (shRNA) knockdown cells. One of two LMP1 shRNA constructs targeting two different LMP1 sites encoded in the Akata EBV strain genome and stably expressed in EBV-infected HK1 cells, shLMP1#1, was able to robustly knock down LMP1 expression under monolayer and ALI culture conditions (Fig. 4a). In comparison with monolayer culture, the induction of the differentiation marker involucrin indicated differentiation of HK1 cells in the 3-week ALI culture period beginning at week 1 (Fig. 4a). Knockdown of LMP1 by shLMP1 clone 1 (shLMP1#1) impaired the induction of the immediate early (Z), early (Ea-D), and late (viral capsid antigen [VCA] p18) EBV lytic proteins (Fig. 4b). A recent study of organotypic raft culture of keratinocytes demonstrated that the differentiation-dependent induction of the immediately early proteins BZLF1 (Z) and BRLF1 (R) is dependent on the expression of LMP1 (11). In agreement with these findings, replica ALI culture experiments also demonstrated that the induction of Z and Ea-D proteins was consistently reduced in LMP1 knockdown HK1 cells (Fig. 4c). Furthermore, the decrease in lytic protein induction in LMP1 knockdown cells correlated with a consistent decrease in extracellular EBV titers (−2.88- to −9.28-fold) in five independent experiments of cells harvested from week 2 ALI cultures, as measured by the green Raji unit assay (Fig. 4d). These data support the growing evidence that LMP1 is required for efficient productive EBV infection in differentiating epithelia (11).

## DISCUSSION

**FIG 5:**
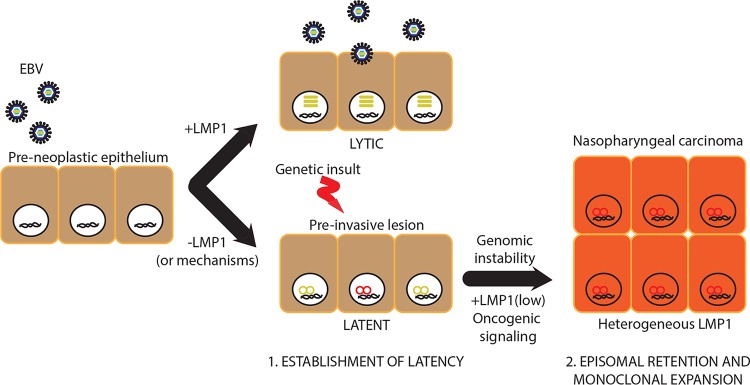
Working model for LMP1 in EBV pathogenesis in a developing nasopharyngeal carcinoma.

The central hypothesis for LMP1 as an oncogenic signaling protein is that it is consistently expressed and detected in NPC tumors (10, 13). Several lines of evidence are consistent with the alternative hypothesis that suppression of LMP1 expression or a mechanism(s) may favor EBV persistence and latency early in NPC oncogenesis. First, although LMP1 can be detected in clonal proliferations of preinvasive lesions, it is rarely detected in preneoplastic nasopharyngeal cells (19, 25). Second, despite EBV clonality, NPC cells show heterogeneous intratumoral LMP1 staining (13, 15). Third, the NPC cell line C666-1, which uniquely retains native EBV infection, expresses low to undetectable levels of LMP1 protein (31). In comparison to the high levels detected in B cell infections, low levels of LMP1 reminiscent of epithelial infection are sufficient to induce oncogenic properties in a number of nonlymphoid cell lines (10, 12, 13). To promote oncogenic signaling and retention of EBV genomes, a low level of LMP1 expression and/or suppression of an LMP1 mechanism(s) distinct from oncogenic signaling may be an important selection criterion for the potentially rare transforming event in an infected cell. A proposed working model for the role of LMP1 in EBV pathogenesis in a developing NPC is illustrated in Fig. 5, in which initiating events leading to a subset of cells expressing LMP1 provide the oncogenic stimulus while cells suppressed for LMP1 expression or an LMP1 mechanism(s) provide the latent reservoir. In comparison to other human tumor viruses, such as human papillomavirus and Merkel cell polyomavirus, which are characterized by integrated viral genomes in tumor cells, EBV-associated cancers, including NPC, maintain EBV genomes as extrachromosomal episomes. In this study, it is unclear if the subsets of cells that retain EBV genomes grown in the absence of selection are integrated, as Southern blotting was not sensitive enough to detect integration by the terminus assay (data not shown). A prior study in AdAH cells supports the possibility that epithelial cells that retain EBV infection are not integrated, as the ability to retain EBV genomes was not a heritable trait (6). The conclusion that LMP1 contributes to efficient permissive replication in differentiating epithelia is in agreement with a recent finding using a similar differentiation-dependent lytic reactivation model in keratinocyte raft cultures (11). There is therefore accumulating evidence that LMP1, also expressed and induced in the lytic cycle, may be perturbed in early preneoplastic cells to establish a latent infection. The phenomenon of an unstable infection also occurs in endothelial cells infected with Kaposi sarcoma-associated herpesvirus (32). Thus, the switch to a persistent and latent infection in preneoplastic cells is likely a common driver event for cancers associated with oncogenic gammaherpesviruses.

The LMP1 protein is not known to possess intrinsic enzymatic activity, and although it is possible that LMP1 directly interferes with the retention or propagation of nuclear EBV genomes, it is equally likely to exert effects through modulation of cellular signaling pathways (13). One consideration is that although LMP1 is an oncogenic protein, high levels of LMP1 have also been ascribed to cytostatic effects (33). It is plausible that the loss of EBV genomes observed in the absence of selection can be attributed not only to inefficient EBV genome retention but also to a competitive growth advantage for cells that have lost EBV infection. In this case, it is anticipated that cells expressing high levels of LMP1 will result in a more rapid loss of EBV genomes as more uninfected cells accumulate. However, 293 cells stably expressing high levels of LMP1 (B958 and China1) did not exhibit accelerated EBV genome loss compared to cells infected with wild-type EBV-BAC expressing physiological levels of LMP1 (Fig. 1a, c, and d). This suggests that the cytostatic effects of LMP1 are not markedly apparent in the 293 cell infections analyzed in this study.

A major challenge in understanding the role of EBV infection in epithelial cancer is the difficulty in establishing EBV-infected cell lines, which stands in contrast to the EBV-immortalizing properties of B lymphocytes. The work described in this study has important implications for understanding the viral determinants of EBV pathogenesis but may also impact efforts to develop improved EBV culture models in epithelial cells.

## MATERIALS AND METHODS

### Cell culture and stable cell lines.

The 293 cell lines infected with wild-type EBV-BAC (2089 clone) or EBV-BAC mutants (ΔLMP1, LMP2A, ΔPXQXT, Y384G mutants) (generous gifts from Wolfgang Hammerschmidt, Helmholtz Zentrum München, Munich, Germany) were maintained with 0.1 mg/ml hygromycin selection as previously described (23). The HK1 NPC cell line infected with the EBV recombinant Akata strain (a generous gift from George Tsao, Hong Kong University, Hong Kong) were maintained with 0.8 mg/ml neomycin selection in RPMI 1640 medium supplemented with 10% fetal bovine serum as previously described (28, 34). The same culture medium was used in the HK1 ALI cultures. Stable cell lines were generated by transduction with retroviral vectors expressing HA-tagged LMP1 (B958 and China1 strains), dominant negative Akt isoform 2 (K181M T309A S474A), IκBα superrepressor (S32A S36A), cyclin D1, shRNAs to LMP1, or a universal nontargeting shRNA sequence and selected with 1 µg/ml puromycin or 500 µg/ml neomycin, as previously described (31, 35, 36). The target sequence for LMP1 in the EBV Akata strain for shLMP1#1 is AACTGGTGGACTCTATTGG, and that for shLMP1#2 is AAGAGACCTTCTCTGTCCACT (29, 36). The Akt2 kinase mutant (K181M T309A S474A) was subcloned from pHA-Akt2 (T309A S474A) by site-directed mutagenesis using two PCR primer pairs, Akt2 MutP-A (5′ GGGTTTGGATCCATGTACCCTTAT 3′)/Akt2 MutP-B (5′ GCAGGATCATCATGGCGTA 3′) and Akt2 MutP-C (5′ TACGCCATGATGATCCTGC 3′)/Akt2 MutP-D (5′ GGTTTGAATTCTCACTCGCGAAT 3′), at the BamHI and EcoRI sites of the pBabe vector. The pBabe-CyclinD1 and pHA-Akt2 (T309A S474A) vectors were obtained from Addgene (catalog numbers 9050 and 60128).

### DNA extraction and quantitative PCR.

Frozen cell pellets were extracted for total DNA with the GeneJET genomic DNA purification kit with RNase treatment (Thermo Scientific). Quantitative PCRs were assembled with the Maxima SYBR Green qPCR master mix kit (Thermo Scientific), by using the recommended cycle conditions with ROX as a passive reference dye and 20 ng genomic DNA as the template. Primer sequences for qPCR were as follows: for qgGAPDH, 5′ CTGGGCTACACTGAGCACC 3′ and 5′ AAGTGGTCGTTGAGGGCAATG 3′, and for qBALF5, 5′ GAGCGATCTTGGCAATCTCT 3′ and 5′ TGGTCATGGATCTGCTAAACC 3′ (37). Primer concentrations were optimized to 0.5 µM for detection of GAPDH and 0.3 µM for BALF5 genes and evaluated for a singular PCR product of the correct size by melt curve and agarose gel analysis. Reactions were performed on the StepOnePlus instrument (Applied Biosystems) and analyzed with StepOne software (v2.3). Statistical analysis was evaluated from triplicate technical replicas. Values were normalized to those for GAPDH, and relative fold change (designated RQ in the StepOne software) was calculated by the ΔΔ*C*_*T*_ comparative method (where *C*_*T*_ is threshold cycle). Error bars were calculated with StepOne software and represent the minimum and maximum normalized fold change values (RQ min, RQ max) set to 95% confidence levels.

### Statistics.

Quantitative PCR plots were analyzed in GraphPad PRISM v7. The qPCR plots were analyzed by the run test for departure from linearity and used to determine that the linear regression model is applicable for statistical analysis. Slopes calculated from linear regression were compared for statistical differences between conditions with and without selection. *P* values below 0.0001 were considered significant.

### EBER-ISH.

EBER *in situ* hybridization (EBER-ISH) was performed on paraformaldehyde-fixed cells (4% paraformaldehyde with 5%, vol/vol, acetic acid) using the EBER PNA probe kit (Dako) according to the manufacturer’s recommendations and analyzed on the BD Accuri C6 instrument by flow cytometry (UPMC cytometry shared facility at the University of Pittsburgh). HK1 cells infected with the recombinant EBV-Akata strain (HK1 EBV) not incubated with the EBER PNA probe and HK1 uninfected cells incubated with the EBER PNA probe were used as negative controls and used to set the positive staining gate (G1).

### Immunoblot analysis.

Whole-cell lysates were prepared in radioimmunoprecipitation assay (RIPA) buffer supplemented with 1 mM phenylmethylsulfonyl, 2 mM activated sodium orthovanadate, a 1:100 dilution of protease, and phosphatase inhibitor cocktails (Sigma), and immunoblot analysis was performed as previously described (36). Briefly, protein concentration was determined with the Bio-Rad DC protein assay. Equal amounts of protein were separated by denaturing SDS-PAGE and transferred to nitrocellulose membranes. Immunoblot analysis was performed using the following antibodies: HSC70, EBV zebra (Z), EBV Ea-D (Santa Cruz Biotechnology), EBV viral capsid antigen (VCA) p18 (Invitrogen), the HA tag (Covance), LMP1 (S12 clone hybridoma tissue culture supernatant, 1:10 to 1:50), and involucrin (Sigma). Membranes were detected with horseradish peroxidase-conjugated secondary antibodies (Jackson ImmunoResearch) and developed with a WesternBright enhanced-chemiluminescence (ECL) detection kit (Advansta).

### ALI cultures.

Single-cell suspensions of HK1 cells (5 × 10^5^ cells) were seeded on collagen-coated polyester membrane transwells (0.4-µm pore size, 12-mm diameter; Corning) in 0.5 ml apical and 1 ml basolateral culture media. Once a confluent monolayer had formed as determined by visual inspection by phase microscopy (1 to 2 days), the apical medium was removed and monitored for leakage from the basolateral compartment to establish an air-liquid interface. Cells at the air-liquid interface were refed twice a week from the basolateral surface and cultured for an additional 3 weeks. EBV was harvested from the apical surface by washing cells in media or phosphate-buffered saline (PBS), and cell lysates were collected in RIPA buffer for immunoblot analysis.

### EBV titers.

EBV titers were determined from week 2 ALI cultures. The titer of infectious EBV encoding the GFP marker in the nonproducer Raji Burkitt lymphoma cell line was determined by using the green Raji assay as previously described (29). Titers of serial dilutions of clarified supernatants harvested from ALI cultures of 0.5 × 10^4^ to 1 × 10^4^ Raji B cells were determined in the presence of 20 nM tetra-decanoyl phorbol-13-acetate (TPA) and 5 mM sodium butyrate. Titers were calculated by counting GFP-positive cells as green Raji units (GRU). Each sample was subjected to triplicate titrations to determine an average number of GRU per ml.

### Southern blotting.

Southern blotting with a biotinylated LMP1 DNA probe was performed according to previously published protocols with the following modifications (38). BamHI-digested genomic DNA separated on a 0.7% agarose gel was transferred onto Biodyne B nylon membranes (Thermo Scientific), and the membranes were cross-linked (UV Stratalinker 1800) and hybridized with the biotinylated LMP1 probe (50 ng/ml) in hybridization buffer (6× SSC [1× SSC is 0.15 M NaCl plus 0.015 M sodium citrate], 0.5% SDS, 5× Denhardt’s solution, 100 μg/ml denatured sheared salmon sperm DNA) at 55°C overnight. After three washes (0.1× SSC, 0.1% SDS) at 55°C for 30 min, the blots were blocked, incubated with streptavidin-horseradish peroxidase, and developed with the ECL substrate according to the instructions of the Chemiluminescent Nucleic Acid Detection Module (Thermo Scientific). The biotinylated LMP1 probe was synthesized by PCR amplification using 10 ng of EBV B958-BAC DNA as the template, with primers 5′ GCATGAGAGCAAAGGAATAG 3′ and 5′ TAGCCGCCCTACATAAGCCTCT 3′, 1.25 units of DreamTaq DNA polymerase (Thermo Scientific), 0.25 mM each of dATP, dCTP, and dGTP, and a ratio of 60% unmodified dTTP to 40% biotin-16-dUTP (Roche). Random-prime synthesis and radiolabeling of a linearized Xho1a (1.9 kb) probe and hybridization were performed as previously described (39).
